# Scale Modular Test Platform for the Characterization of PD Measuring Systems Using HFCT Sensors

**DOI:** 10.3390/s24051363

**Published:** 2024-02-21

**Authors:** Eduardo Arcones, Fernando Álvarez, Abderrahim Khamlichi, Fernando Garnacho

**Affiliations:** 1 Department of Ingeniería Eléctrica, Universidad Politécnica de Madrid, Ronda de Valencia 3, 28012 Madrid, Spainfernando.garacho@ffii.es (F.G.); 2 FFII-LCOE (Laboratorio Central Oficial de Electrotecnia), C/Eric Kandel 1, 28906 Getafe, Spain

**Keywords:** partial discharges, insulation testing, condition monitoring, performance evaluation, power system modeling, model-driven development, model checking, sensor phenomena and characterization

## Abstract

Today, online partial discharge (PD) measurements are common practice to assess the condition status of dielectrics in high-voltage (HV) electrical grids. However, when online PD measurements are carried out in electrical facilities, several disadvantages must be considered. Among the most important are high levels of changing electrical noise and interferences, signal phase couplings (cross-talk phenomena), and the simultaneous presence of various defects and difficulties in localizing and identifying them. In the last few decades, various PD-measuring systems have been developed to deal with these inconveniences and try to achieve the adequate supervision of electrical installations. In the state of the art, one of the main problems that electrical companies and technology developers face is the difficulty in characterizing the measuring system’s functionalities in laboratory setups or in real-world facilities, where simulated or real defects must be detected. This is mainly due to the complexity and costs that the laboratory setups entail and the fact that the facilities are permanently in service. Furthermore, in the latter scenario, owners cannot assign facilities to carry out the tests, which could cause irreversible damage. Additionally, with the aforementioned installations, a comparison of results over time in various locations is not possible, and noise conditions cannot be controlled to perform the characterizations in a correct way. To deal with the problems indicated, in this article, an affordable scale modular test platform that simulates an HV installation is presented, where real on-site PD measuring conditions are simulated and controlled. In this first development, the HV installation comprises a cable system connected at both ends to a gas-insulated substation (GIS). As the most common acquisition technique in online applications is based on the placement of high-frequency current transformer (HFCT) sensors in the grounding cables of facilities, the test platform is mainly adapted to carry out measurements with this type of sensor. The designed and developed test platform was validated to assess its features and the degree of convergence with a real installation, showing the convenience of its use for the appropriate and standardized characterization of PD-measuring systems.

## 1. Introduction

PDs are partial short circuits in dielectrics, formed by the ionization processes of low energy, where inhomogeneous electric fields are present. When an HV asset has an insulation defect, the presence of PD activity is common. When PD occurs, repetitive pulsating signals of very short duration are generated. Several studies have demonstrated that PD activity is closely related to the degradation of the insulation materials of HV electrical grids [1,2,3]. PD measurement makes it possible to detect insulation defects before dielectric breakdown due to a short circuit. Dielectric breakdowns cause risks to individuals, material damage, losses of power supply, and increased operating costs. Online PD measuring is gaining special attention because it makes the effective detection of defects in installation assets possible [4,5,6,7]. The main advantage of online measurements is that they are performed when the facilities are in normal operation. To appropriately detect PD activity and perform accurate diagnosis, various measuring applications based on electromagnetic methods have been and continue to be developed [8,9,10,11,12]. These applications usually have specific functionalities, such as background noise filters to improve the sensitivity and PD source discrimination, as well as location and defect identification tools [3,4,13,14]. They also have the capacity to generate assisted or automatic alarms when a critical defect is identified [15]. On many occasions, the diagnosis results show varying degrees of success depending on the technology implemented, the effectiveness of their functionalities, and the training and expertise of the technical analysts.

When the benefits of using a particular functionality are presented, complex laboratory setups with artificial defects in a certain asset or in test cells are usually used [4,6,14]. On other occasions, for this purpose, on-site case studies in real installations have been presented [3,7,11,15]. The laboratory setups required are complex and expensive, and can only be used in specific locations. Moreover, on-site installations cannot be generally used for this purpose due to their lack of availability and the risks involved when tests are performed at the location. In both cases, it is not possible to make a comparison of the results over time in different emplacements regarding various technologies, and the noise conditions during the measurements cannot be adequately controlled. Currently, electrical companies and technology developers are requesting a technical solution that enables the characterization of the measuring system’s functionalities in a reproducible manner, without the requirement of using specific laboratory setups or on-site electrical facilities.

To facilitate the performance of characterization tests, in [16,17], small-scale systems with isolation defects generated in individual insulation elements or test cells were developed. The implementation of the above systems is useful, since PD activity from several defects can be simultaneously generated in portable structures. However, the previous setups have the following disadvantages:

- HV application is required, and the PD is generated at the measuring point. That is, the measured pulses are not representative of those measured on site;
- Given the stochastic nature of the PD generated over time, the performance of reproducible or standardized tests with these scale systems is not possible. Thus, the realization of intercomparisons among various technologies is not possible;
- The noise influence during measurements cannot be adequately controlled;
- The physical conditions for PD measurements in real, three-phase installations are not reproduced. Thus, a consideration of technical aspects such as phase coupling and pulse attenuation, distortion, and reflection is not possible. This implies that some functionalities, such as those developed for affected phase identification, defect location, or defective element recognition, cannot be characterized;
- The measurement conditions regarding PD acquisition significantly differ from those on site.

To address the first three previously noted disadvantages in [18,19,20,21], a solution based on the controlled generation of PD time series is presented. An analog generator reproduces artificial PD and noise signals or signals previously measured in a laboratory setup or on site, with the additional advantage of not being necessary in the generation of HV. However, although this solution is useful, the capabilities of an analog PD generator are insufficient to overcome the last two disadvantages. To deal with these disadvantages, a specific scale modular test platform was developed to simulate reproducible real physical measuring conditions and is presented in this paper. The injection of an analog time series in the test platform initiates the opportunity to address all the technical aspects required, such as noise influence, sensor coupling and signal transmission, attenuation, distortion, and reflection. Furthermore, the use of the test platform precludes having to carry out HV tests in complex laboratory setups or on-site facilities. In addition, the use of the generator with the test platform inside a shielded chamber allows for the realization of evaluation tests under controlled noise conditions.

The next section focuses on the presentation of the modular test platform. Section 3 assesses its validation, showing its degree of convergence with a real HV installation. Lastly, Section 4 is dedicated to the conclusions. In this last section, the benefits of using the test platform are indicated.

## 2. Scale Modular Test Platform Design

For the appropriate characterization of PD-measuring systems, the configuration of the test platform must be representative of a three-phase power installation and simulate its physical online and offline measuring conditions.

To achieve an adequate physical model, various concepts must be considered, such as the model layout configuration and technical features related to the following aspects: PD injection points where the insulation defects are reproduced, the waveform of the injected signals in simulating the defects, the measuring technique, and the measuring points for PD acquisition.

**Figure 1:**
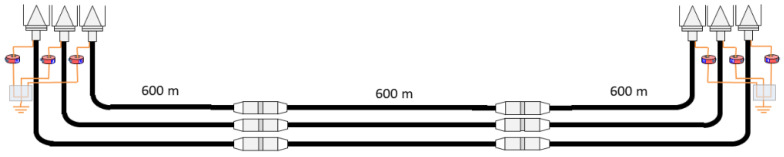
Layout of the HV distribution line considered for reproduction in the test platform.

In the first approach, for simplicity, the design of the platform is focused on the simulation of an underground HV distribution line. The three-phase line has two straight joints (see Figure 1), and it is connected at both ends to a GIS substation.

**Figure 2:**
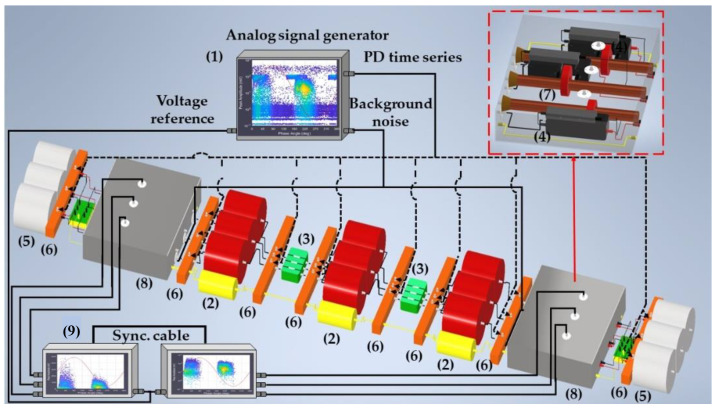
Layout of the scale modular test platform developed for the characterization of the PD-measuring systems’ functionalities.

For the characterization tests’ implementation, the test platform comprises the following elements (see Figure 2):

- Analog signal generator (ASG) subsystem (1): In this element, PD and electrical noise signals are generated for the measuring systems’ characterization. These signals are of the same nature as those present in the power grids;
- Functional scale module subsystem, consisting of the insulated three-phase cable elements (2), straight junction chambers (3), cable-GIS connection elements (4), and GIS modules (5);
- Defect injection distributed subsystem (6), which is used for the simulation of PD sources associated with insulation defects. The PD time series can be injected into the following elements of the installation: GIS compartments, cable terminals, and cable joints;
- Distributed HFCT sensors subsystem (7), which is used to carry out PD measurements in two proposed positions of the installation;
- Noise injection subsystem (8), which is used for the appropriate reproduction of the same background electrical noise-measuring conditions of a real installation. Within this subsystem are the cable–GIS connection elements (4) and the HFCT sensors (7);
- A measuring subsystem (9), with at least a three-channel acquisition unit per measuring point.

In the design process, the following aspects were considered to achieve a feasible test platform.

- Selection of an appropriate cable for the simulation of the power coaxial cable of a real installation. The best option was to choose a commercial signal coaxial cable that was cost-effective, lightweight, and not bulky, and with adequate impedance matching, attenuation behavior, and propagation speed;
- Once the signal cable was selected, for the reproduction of the traveling wave behavior, the technical features of cable attenuation, signal reflection, and propagation speed were considered in detail;
- Furthermore, the signal behavior in the measuring points needed to be adequately reproduced. In this way, impedance matching in the cable–GIS connection points (border points) was undertaken in two steps to reproduce the same conditions as in an HV installation;
- In addition, the method of generating and injecting insulation defects and electrical noise needed to be considered so as to simulate the same measuring conditions as in an HV installation;
- In the final stage of the design, it was necessary to validate the test platform. For this purpose, various measurements were carried out in a real HV cable and setup and in the first test platform prototype. The validation study was reinforced with simulations performed using software tools.

All these issues are treated in detail in the following sections.

The design of the test platform used to simulate the real installation considered the reference values of the parameters of the involved assets. These values were obtained with experimental frequency sweeps in laboratory tests under standard conditions (temperature of 20 °C) and subsequently compared with those provided by the manufacturers at the same temperature. The platform validation tests were performed at this temperature. Thus, proper simulation and verification were achieved.

For result-comparison purposes, when characterization tests were performed, the same temperature was required to be set in order to maintain the same measuring conditions and properties in the test platform.

### 2.1. Selection of a Commercial Signal Coaxial Cable

With a view to developing a commercially viable test platform, its price, weight, and dimensions must be adequate. In addition, in the points where the insulation defects are simulated, impedance matching with the ASG must be performed. This last technical requirement is necessary to avoid signal distortion and incorrect Q_IEC_ charge values in the measurements. Since the standardized output impedance of ASGs is 50 Ω, the selection of a signal cable with a characteristic impedance of 50 Ω is highly recommended.

Furthermore, the signal propagation speed of the selected cable should be as close as possible to that of the HV cable to be simulated. The cable to be simulated is of 66 kV, 1200 mm^2^ aluminum conductor and has a 9 mm-thick, cross-linked polyethylene (XLPE) insulation.

The minimum propagation speed for the signal cables referred to in the technical specification MIL-C-17 [22] is 198 m/μs. Thus, as the propagation speed of the HV cable to be simulated is around 169 m/μs, and the most adequate signal cables of the MIL-C-17 regarding this parameter are those with the lowest propagation speed.

Additionally, cable attenuation must be considered. High attenuation values with respect to the characteristic values of HV lines mean that in order to obtain the same levels of attenuation, the effective lengths in the model will be shorter. With a shorter effective equivalent length, the weight, volume, and costs will be reduced in the test platform.

Taking into account the aforementioned indications, after a survey was conducted, the most suitable cables considered for detailed analysis were LLF 240, RG 59, RG 58, and RG 174. The characteristics of these cables were set according to the technical specification MIL-C-17. The features indicated in Table 1 were assessed for these cables under the assumption that each had the same weight. The rating criteria were as follows: 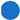 excellent (4 points), 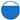 very good (3 points), 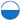 good (2 points), 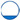 bad (1 point), and 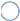 very bad (0 points). According to the scores obtained, the RG 174 cable was deemed the most suitable for use in the implementation of the test platform. This cable contained a 0.14 mm^2^ copper conductor and a polyethylene (PE) insulation 1.5 mm in diameter.

### 2.2. Signal Attenuation Consideration and Initial Estimate of the Adequate Real Line to Be Simulated

To perform accurate diagnoses using PD measurements, the analysis of pulse attenuation with the traveled distance is essential. The attenuation levels in the test platform must be very similar to those that occur in the HV line. To achieve this, the effective length of the RG 174 cable must be defined.

**Figure 3:**
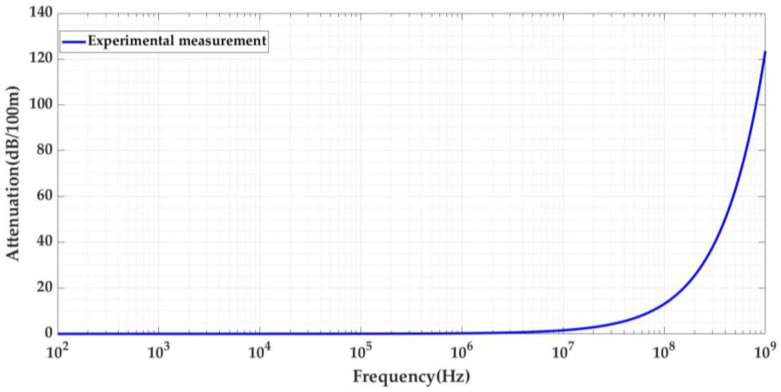
Signal attenuation in 100 m as a function of frequency for the 66 kV XLPE cable.

The signal attenuation is frequency-dependent, and to characterize how it affects PD pulses, it is necessary to establish the traveled distance up to which attenuation below a certain frequency can be considered negligible. Furthermore, it is also necessary to determine the reference distance traveled above which the spectral components of interest can be considered negligible. For this purpose, the attenuation curve of the 66 kV XLPE cable was analyzed from 100 Hz to 1 GHz (see Figure 3).

**Figure 4:**
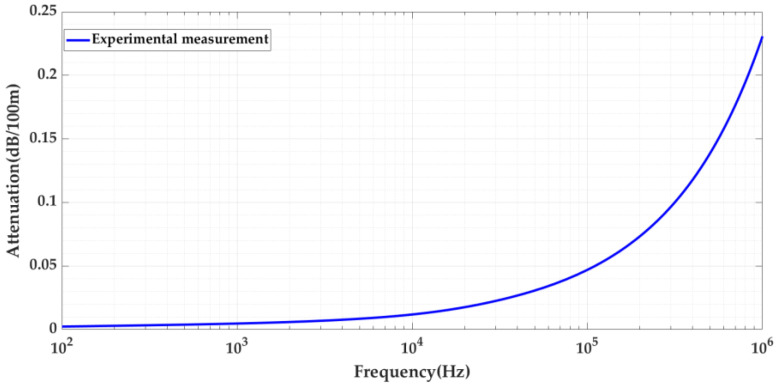
Detail of the signal attenuation from 100 Hz to 1 MHz for the 66 kV XLPE cable.

The graph depicts 100 Hz to 1 MHz (see Figure 4), and so it can be observed that up to 100 kHz, the attenuation of this cable is slightly below 0.05 dB/100 m. This means that to attenuate 1 dB (i.e., to lose 10% of the pulses energy) for frequencies up to 100 kHz, the pulses have to travel more than 2000 m. Therefore, below 2 km for frequencies up to 100 kHz, the attenuation effect over distance can be considered negligible. Thus, when comparing the attenuation of both cables, if the maximum traveled distance simulated in the test platform is less than 2 km, the lower frequency limit to be considered is 100 kHz.

The study described in this paper was focused on PD acquisition using HFCT sensors; consequently, the higher frequency of interest can be considered as being up to 100 MHz. For frequencies above 100 MHz, the attenuation is greater than 13 dB/100 m, i.e., the pulses are attenuated by more than 78%. When the pulses travel more than 153 m, the attenuation will be greater than 90%. Thus, if the distance to be simulated in the test platform between an insulation defect and any measuring point is greater than 153 m, the frequency content of the traveling pulses can be considered negligible above 100 MHz.

In the characterization of attenuation, according to these previous considerations, the frequency interval of interest could be set as between 100 kHz and 100 MHz.

**Figure 5:**
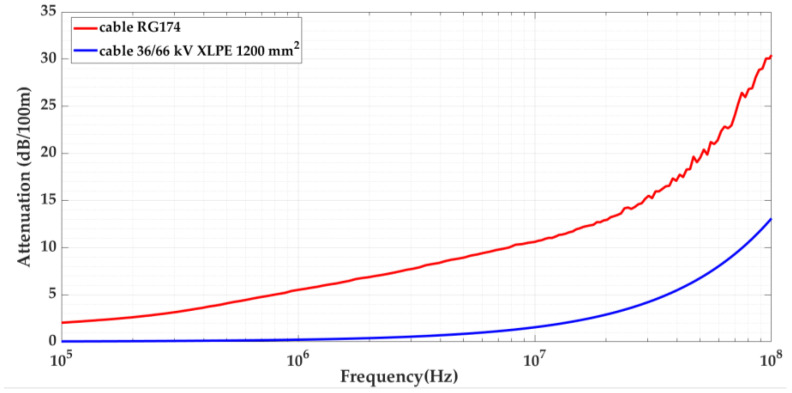
Signal attenuation at 100 m as a function of frequency for the 66 kV XLPE and RG 174 cables in the range from 100 kHz to 100 MHz.

The attenuation analysis over distance was also performed for the RG 174 cable. Figure 5 shows the attenuation curves for both cables. The attenuation curves shown in Figure 3 and Figure 5 were obtained experimentally by performing a frequency sweep for the two cables (66 kV and RG 174).

As the attenuation curves are different, it is necessary to find the equivalent length for the RG 174 cable that will obtain the same attenuation as that in the real cable of 66 kV. To find this equivalent length, for each frequency *f*, the amplitude of a pulse must be the same when traveling a distance *L_MT_* over the cable of 66 kV and its equivalent distance *L_RG_*_174_ over the RG 174 cable; see Equation (1).

(1) $${v(f,L_{MT})}_{cable\;MT}={v(f,L_{RG174})}_{cable\;RG174}$$

If we replace in each term of Equation (1), Equation (2) [23] describing the propagation distance, where *γ*(*f*) is the propagation coefficient and *V_o_* the initial pulse voltage for each frequency, the relationship between the two cable lengths can be expressed via Equation (3).

(2) $$v\left(f,L_{x}\right)=V_{o}\left(f\right)\cdot e^{-\gamma (f)\cdot L_{cable}}$$

(3) $$\frac{L_{MT}}{L_{RG174}}=\frac{\gamma_{RG174}(f)}{\gamma_{MT}(f)}$$

The attenuation in decibels (dB) is determined by Equation (4). The propagation coefficient *γ*(*f*) of each cable is obtained first by substituting Equation (2) into Equation (4) [24] and then replacing in Equation (5), the values of ΔdB taken from Figure 5 for a cable length (*L_Cable_*) of 100 m.

(4) $$\Delta dB\left(f,L_{cable}\right)=-20\cdot log\left(\frac{v\left(f,L_{cable}\right)}{V_{o}\left(f\right)}\right)$$

(5) $$\Delta dB\left(f,L_{cable}\right)=8.68\cdot \gamma (f)\cdot L_{cable}$$

**Figure 6:**
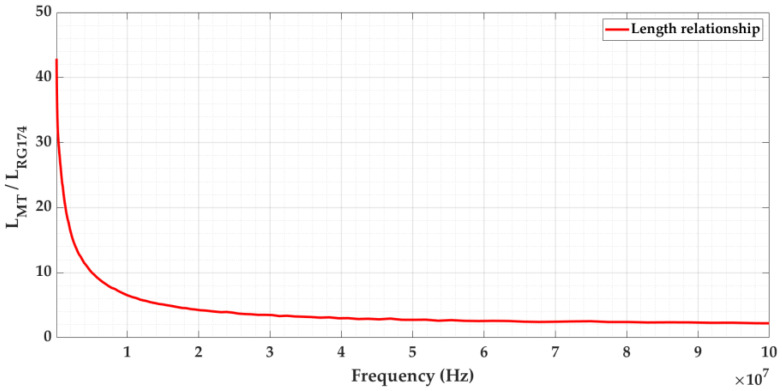
Relationship between the length of both cables in the frequency range from 100 kHz to 100 MHz.

The relationship between the length of both cables as a function of frequency (see Figure 6) is obtained using Equation (3).

As shown in Figure 6, the length ratio as a function of frequency shows an inverse exponential behavior. Therefore, in the first approach, obtaining a constant value to establish an equivalence between both lengths is not possible. In the second approach, by iterating in Equation (6) the length ratio (*Γ*), an average value can be calculated for it.

The average ratio is obtained for the value at which the lowest attenuation at low frequencies is compensated by the highest attenuation at high frequencies. Starting from an initial value of *Γ*, in each iteration, the difference in areas between the exponential curve of ratio $\left(\frac{L_{MT}}{L_{RG174}}\right)$ and the line of the average value *Γ* is calculated before and after the cutoff frequency between both $\left(f_{\Gamma }:\frac{L_{MT}}{L_{RG174}}\left(f_{\Gamma }\right)==\Gamma \right)$. The final ratio value at which the iteration ends is then the one that makes both area differences equal. With this value, the pulses traveling along both cables end up being as similar as possible. This equivalence can be adapted to any type of cable being simulated in the test platform.

(6) $$\int_{100\;KHz}^{f_{\Gamma }\;\;:\;\frac{L_{MT}}{L_{RG174}}\left(f_{\Gamma }\right)==\Gamma }\frac{L_{MT}}{L_{RG174}}\left(f\right)-\Gamma \;df=\int_{f_{\Gamma }\;\;:\;\frac{L_{MT}}{L_{RG174}}\left(f_{\Gamma }\right)==\Gamma }^{100\;MHz\;}\Gamma -\frac{L_{MT}}{L_{RG174}}\left(f\right)\;df$$

For the cables under study, the ratio value obtained was 5.8. When applying this value, the waveform and frequency spectrum of the traveling pulses in both cables converged by more than 85%. The degree of convergence obtained is presented in Section 3.1.

The relationship obtained establishes that 100 m of the 66 kV cable being simulated is equivalent to an effective length of 17 m of the RG 174 cable used in the test platform.

The signal attenuation characterization described in this section is useful for deriving an initial estimate of the adequate real line to be simulated. Thus, it is desirable, for one, that in this line the total length traveled by a pulse be less than 2 km, and for another, that the distance traveled by a pulse between a PD source and any measuring point be greater than 153 m.

### 2.3. Signal Reflection Consideration in the Cable System and Line Length Estimate

**Figure 7:**
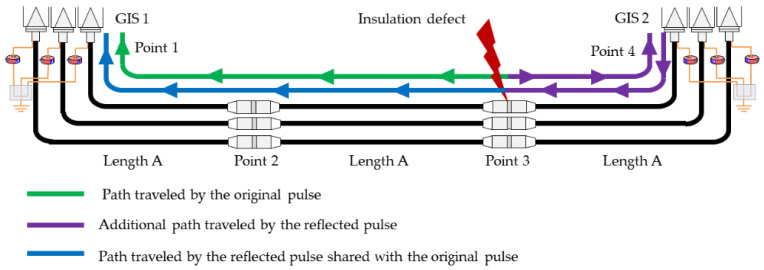
Modeled line with cable sections of length A and the pulses’ traveling paths when a defect is simulated in point 3.

To simulate the same measurement conditions as in a real HV installation, the effect of the signal reflection on the impedance changes must be considered. For the line shown in Figure 1, these impedance changes take place at the cable–GIS connections points. In straight joints, if the accessories are correctly assembled, there are no impedance changes that give rise to appreciable reflections. In real cable systems, as the distances are generally long, the waveform of the measured pulses is not affected by superposition with pulses coming from reflections. This same scenario must be maintained in the test platform. The duration of the PD pulses measured with HFCT sensors in HV lines generally does not exceed 1 μs. Thus, if the time delay of the reflected pulses with respect to the original ones is greater than 1 μs, we can infer the absence of overlapping between them. Considering the signal propagation speed of the RG 174 cable (198 m/μs) and of the cable with 66 kV (169 m/μs), a time of 1 μs corresponds to additional distances traveled by the reflected pulses of 198 m and 169 m, respectively. In this way, to avoid overlapping, in the worst case, the additional distances traveled must be greater than these distances. For the modeled line in the test platform (see Figure 7) and the measurements performed at point 1, the additional distances are indicated in Table 2, with the presence of defects at points 1, 2, 3, and 4 being considered. The reference cable section length A was considered for the test platform. If the measurements were to be performed at point 4, the results would be equivalent since the line has a symmetrical configuration.

It can be verified that when the measurements are performed in point 1, the worst case—that is, the shortest distance (2A)—is obtained when there is a defect in point 3 (second joint). Figure 6 shows the pulses’ traveling paths when there is a defect in this point. The violet line represents the additional path (2A) traveled by the reflected pulses.

Therefore, to ensure the absence of pulse overlaps, the cable section length A set for the test platform is 100 m, with the distance 2A (200 m) being greater than the minimum required (198 m). In the previous section, it was established that the length ratio between the RG 174 cable and the cable of 66 kV is 5.8; thus, the 100 m section selected in the test platform corresponds to 580 m in the HV installation. When using this distance in the real installation, there is no overlap in the measured and reflected pulses. The complete cable systems’ lengths, considering that they are formed from three cable sections (see Figure 7), are 300 m and 1740 m, respectively.

### 2.4. Signal Propagation Speed Consideration

Another feature to consider in the test platform design is the signal propagation speed (*V_p_*). In PD diagnosis, this parameter is used for defect localization. When an online measurement is performed using HFCT sensors with the distance between them (*L*) and the signal propagation speed (*V_p_*) being known, the localization of the PD sources is possible. When applying time-of-flight analysis to the measured pulses [4], the equations below are used. For each phase, a pulse detected by one sensor is associated with another detected by a consecutive one only if the time delay between their arrival times Δ*t* is less than the signal propagation time between the measuring points *t_w_* (defined in Equation (7)).

(7) $$t_{w}=\frac{L}{V_{p}}$$

With the parameters Δ*t*, *t_w_*, and *L* being known, the location of a pulse source *x*(Δ*t*) can be determined using Equation (8).

(8) $$x\left(∆t\right)=\frac{L}{2}\cdot \left[1-\left(\frac{∆t}{t_{w}}\right)\right]$$

For the characterization of the functionalities of measuring systems used for defect localization, the main technical requirement is that the signals generated in the PD sources arrive with the expected delay at the sensors position.

To simulate a real 66 kV cable 1740 m in length for PD source localization purposes, the propagation speed of an RG 174 cable 300 m in length must be redefined and determined as follows. As the propagation speed of the RG 174 cable is 198 m/μs, the signals take 1.5 μs to travel the 300 m. This is the real propagation time and cannot be modified. Thus, as the length to be simulated is 1740 m, to maintain the calculated propagation time, a new fictitious propagation speed parameter is set for the RG 174 cable. This new parameter is called the equivalent propagation speed (*V_pe_*), and its value is given by Equation (9).

(9) $$V_{pe}={V_{p}}_{RG174}\cdot \frac{L_{HV}}{L_{RG174}}$$

where *V_pRG_*_174_ and *L_RG_*_174_ are the propagation speed and length of the RG 174 cable, respectively, and *L_HV_* is the length of the HV cable.

The equivalent propagation speed value obtained, *V_pe_* = 1164.2 m/μs, must be considered for the estimation of the defects’ locations in the test platform.

### 2.5. Impedance Matching and Measuring Conditions

To simulate the same pulse behaviors, the effect of impedance change at the cable–GIS connections must be the same in the test platform and in the HV installation. In addition, the measuring conditions in the grounding connections must also be the same. To fulfil the above, a study was carried out in two stages. In the first one, the coaxiality between the cable and GIS was maintained up to their junction point. This consideration enabled an estimate of the GIS characteristic impedance and the design of this element in the test platform. In the second stage, the effects of the cable terminal–earth connection were assessed. Via these considerations, the conditions of measuring using HFCT sensors could be properly reproduced in this critical part of the test platform.

#### 2.5.1. GIS Characteristic Impedance and Design

In any real HV installation, the cable and GIS impedances are always defined. For the estimate of GIS impedance in the test platform, the impedance value of the selected RG 174 (50 Ω) cable must be considered. In the estimation process, a condition is imposed whereby the pulse reflection phenomenon must be the same for the HV line and the test platform. Signal reflection at the boundary points is characterized by the reflection coefficient Г*_z_* [25]. This coefficient depends only on the impedance involved, and its value is determined by Equation (10), with *Z_GIS_* and *Z_cable_* being the GIS and cable impedances, respectively.

(10) $${Г}_{z}=\frac{Z_{GIS}-Z_{cable}}{Z_{GIS}+Z_{cable}}$$

**Figure 8:**
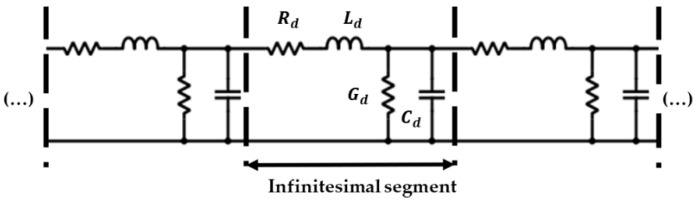
Layout of a transmission line composed of infinitesimal parameters.

Considering the cable and GIS as transmission lines composed of infinitesimal elements (see Figure 8) [26], the value of the previous impedances can be derived from Equation (11) [27], where *R_d_*, *L_d_*, *G_d_*, and *C_d_* are the distributed resistance, inductance, conductance, and capacitance of these elements, respectively.

(11) $$Z=\sqrt{\frac{\left(R_{d}+w\;L_{d}\;j\right)}{\left(G_{d}+w\;C_{d}\;j\right)}}$$

In coaxial geometries, for frequencies above 100 kHz, $R_{d}\ll wL_{d}$ and $G_{d}\ll wC_{d}$, and therefore Equation (11) can be simplified to Equation (12).

(12) $$Z=\sqrt{\frac{L_{d}}{C_{d}}}\;$$

In a real HV line, for the characteristic values of the 66 kV cable and the 66 kV GIS considered (*L_d cable_* (160 nH), *C_d cable_* (211 pF), *L_d GIS_* (203 nH) and *C_d GIS_* (67 pF)), the cable and GIS impedances are 28 Ω and 55 Ω, respectively.

The values of the previously distributed parameters were obtained from the theoretical and practical development presented in Appendix A of this article. Through Equation (10), the reflection coefficient at the boundary point can be inferred to be 0.32. To simulate the behavior of the reflected pulses in the test platform, the same reflection coefficient must be set, and thus impedance matching between the cable and GIS is required. As such, as the RG 174 test platform cable is 50 Ω, the GIS impedance must be calculated using the following equation:

(13) $$Z_{GIS\;platform}=Z_{RG174}\cdot \frac{1+{Г}_{z}}{1-{Г}_{z}}$$

The resulting value of *Z_GIS platform_* to be considered in the design of the GIS was 97 Ω. To achieve an economically viable prototype, an RG 62A coaxial cable of 93 Ω was used to simulate this element of the test platform. This is the cable that best fits the 97 Ω required. The difference between the target reflection coefficient (0.32) and the one achieved with 93 Ω (0.3) is 5%. With this slight deviation, this approach can be considered as yielding an adequate simulation of the reflected pulses.

To simulate the pulses’ behavior when they propagate through the GIS, an equivalent length was established for the RG 62A cable with the same propagation speed as that in a real GIS. As the pulse propagation speed in the 66 kV GIS to be simulated is similar to that of light (*V_p_* = 300 m/µs) and as the propagation speed of the RG 62A cable is 250 m/µs, its length must be 1.2 times shorter than that of the simulated GIS. Assuming that the 66 kV GIS is 10 m long, the length required for the RG 62A cable will be 12 m. As the lengths of the real and simulated GIS are very short, the signal attenuation phenomena can be considered negligible when the pulses travel through this coaxial media.

The degree of pulse reflection in the epoxy resin spacers of the GIS compartments is negligible when the wavelength (λ) of the signals is 10 times longer than the length of these spacers [28], which does not exceed 20 cm. Thus, for wavelengths longer than 2 m, in applying Equation (14) and considering a pulse propagation speed in the spacers of 165 m/µs, for frequencies higher than 87 MHz, their reflections can be neglected.

(14) $$f=\frac{V_{p}}{\lambda }$$

For signals with a spectral content greater than 87 MHz, as the characteristic impedance of the spacers (around 60 Ω) [29] is very similar to that of the GIS compartments (around 55 Ω), the reflection coefficient is very low (around 0.04), meaning that in this case, the reflections can again be neglected.

At the output of the GIS, continuity with a cable system is simulated with the same cable–GIS connection elements as at the input. In addition, these modules are connected to a 50 Ω BNC-type impedance in order to ensure the same impedance as that of the RG 174 cable. 

#### 2.5.2. Consideration of Measuring Conditions 

**Figure 9:**
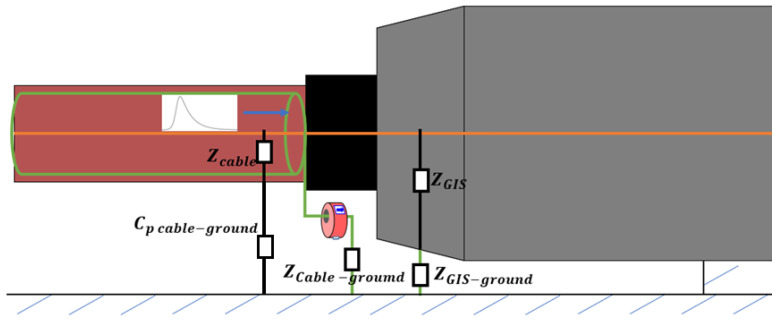
Impedances at the measuring point in the cable–GIS connections.

In real installations, the cable screen is grounded in the connections with the GIS (see Figure 9); thus, at the measurement points, the coaxiality is interrupted. In this case, the waveform of the original pulses coming from the cable is only maintained for the reflected ones that return through it. Consequently, to characterize the signal’s behavior in the grounding cables where the HFCT sensors are located, it is necessary to carry out the following analysis.

To simulate the same measuring conditions in the cable earthing points, the equivalent circuit formed by the impedances at the cable–GIS connection points (GIS–grounding and cable screen–grounding) is considered (see Figure 9).

In this part of the test platform, the impedances are those of the cable (*Z_cable_*), GIS (*Z_GIS_*), cable grounding (*Z_Cable-ground_*), and GIS grounding (*Z_GIS-ground_*), as well as the stray capacitance between the cable and ground plane (*C_p cable-ground_*).

It is assumed that the GIS grounding is close to the cable connection. In this case, the value of the impedance *Z_GIS-ground_* can be considered zero. In addition, it is assumed that in the area close to the cable–GIS junction point, the distance between the cable and the ground plane is large enough (>30 cm) [30,31] to consider the capacitance value *C_p cable-ground_* to be very low. Consequently, the pulses only circulate through the impedance *Z_cable-ground_*.

**Figure 10:**
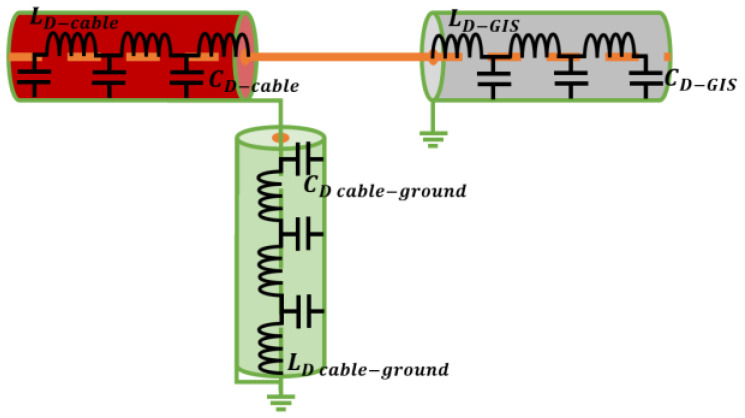
Electrical circuit at the measuring point in the cable–GIS connection.

As the elements used to simulate the cable system (RG 174 cable) and GIS (RG 62 A cable) have already been defined, it is only necessary to determine the value of the grounding impedance (*Z_cable-ground_*) components. For this purpose, the grounding is considered as a transmission line and is studied using the distributed parameter method (see Figure 10).

The circuit shown in Figure 10 was simulated with Simulink for a real 66 kV installation and for the test platform. The values of the distributed cable and GIS parameters considered in the models were obtained from the theoretical and practical development presented in Appendix A. For the grounding cable, a length of 10 m was considered. Taking for the parameters *L_d_*, *C_d_*, and *R_d_* of the real installation, the values of 0.7 µH, 6 pF, and 0.5 mΩ, respectively, very similar pulses can be measured in both installations when for the test platform, these parameters are adjusted to 1 µH, 5 pF, and 0.1 mΩ, respectively. To compare the similarity of the measured pulses, a reference pulse was injected between the conductor and the screen of a two-meter-long cable connected to a GIS. The injected pulse was defined by the inverse double exponential function indicated in Equations (15) and (16) [32], where $V_{p}$ is the pulse peak voltage, $t_{a}$ and $t_{b}$ are the time constants related to its rise and fall time, and $t_{0}$ is the delay from the beginning of the injected signal.

(15) $$V_{o}=V_{p}\cdot \frac{t_{a}+t_{b}}{t_{b}\cdot {\left(\frac{t_{a}}{t_{b}}\right)}^{\left(\frac{t_{a}}{{t_{a}+t}_{b}}\right)}}$$

(16) $$v=\frac{V_{o}}{e^{\frac{t-t_{0}}{t_{a}}}+e^{-\frac{t-t_{0}}{t_{b}}}}$$

**Figure 11:**
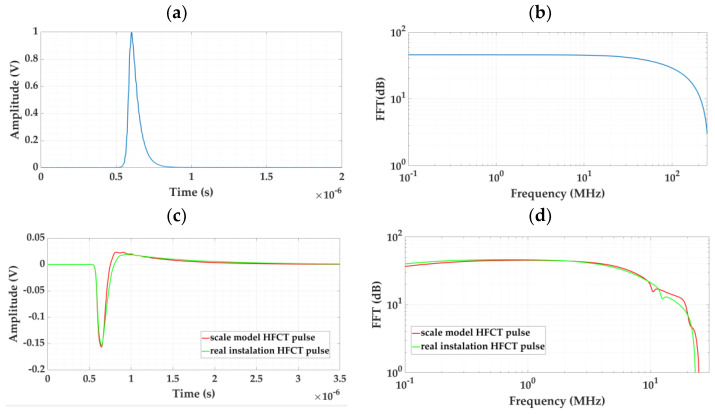
Comparison of a pulse circulating through the cable grounding in the real installation and in the test platform. (**a**) Waveform of the injected pulse, (**b**) frequency spectrum of this pulse, (**c**) waveform of the measured pulses, and (**d**) frequency spectrum of these pulses.

For the same pulse injected in both models (see Figure 11a,b), the waveform and frequency spectrum of the pulses measured are very similar (see Figure 11c,d).

### 2.6. Insulation Defects and Pulsating Noise Simulation

The generation of insulation defects in the test platform can be undertaken in each phase in the two cable terminals, two cable joints, and two GIS compartments (see Figure 2). This makes a total of eighteen injection points. The PD time series corresponding to the defects are generated with the ASG. At each injection point, there is a BNC T-type adapter connected to the main conductor and screen/enclosure of the affected element (cable terminal, cable joint, or GIS). The ASG is wired to the T adapter with an LLF 240 cable of one meter and 50 Ω. This cable was chosen due to its very low attenuation and consequently negligible influence on the injected signals.

**Figure 12:**
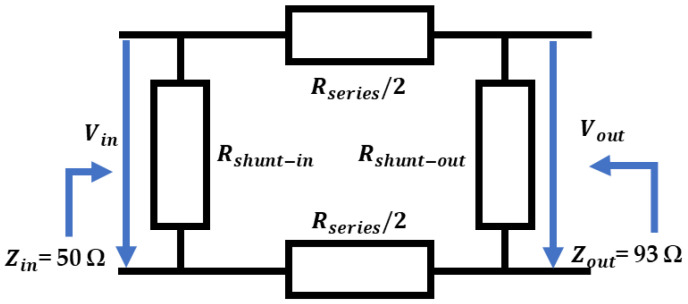
Passive resistance adapter for impedance matching in the GIS injection points.

As the impedances of the ASG and the LLF 240 and RG 174 cables are the same, we see impedance matching in the injection points of the cable terminals and joints. Thus, in these emplacements, there is no pulse reflection. However, as the cable used to simulate the GIS compartment (RG 62-A) is 93 Ω, an impedance matching with the LLF 240 cable is required. This matching is carried out by means of a passive resistance adapter based on the balanced pi attenuator shown in Figure 12 [33]. To achieve the impedance matching, in the first step, the parameter voltage gain (*V_gain_*) is calculated by applying Equation (17), and then the parameter of minimum possible attenuation (attenuation dB) is obtained using Equation (18) [33]. *Z_out_* and *Z_in_* are the RG 62-A (93 Ω) and RG174 (50 Ω) cables’ impedances, respectively. As the value of the minimum possible attenuation is −7.2 dB, the output voltage is 43.4% lower than the input voltage. This reduction is considered in the injection process of the PD time series in the GIS.

(17) $$V_{gain}=\frac{V_{out}}{V_{in}}=\left(\frac{1}{\sqrt{\frac{Z_{out}}{Z_{in}}\;}+\sqrt{\frac{Z_{out}}{Z_{in}}-1}}\right)$$

(18) $$Attenuation\;(dB)=20\cdot log\left(V_{gain}\right)$$

In the second step, the resistances in parallel at the adapter input (*R_shunt-in_*) and output (*R_shunt-out_*) and the two resistances in series (*R_series_*) are calculated with Equations (20)–(22) [33], respectively. *V_out_* is the signal voltage level in the adapter output, which is determined using Equation (19) [33]. For the impedance values of *Z_in_* (50 Ω) and *Z_out_* (93 Ω), the resulting values of *R_shunt-in_*, *R_shunt-out_*, and *R_series_* are 73 Ω, 8 kΩ, and 64 Ω, respectively.

(19) $$V_{out}=\sqrt{\frac{Z_{in}}{Z_{out}}\cdot \frac{1}{10^{\left(V_{gain}\;\cdot \;0.1\right)}}}$$

(20) $$R_{shunt-in}=\frac{\left(Z_{out}\cdot Z_{in}\right)-\left({Z_{in}}^{2}\cdot {V_{out}}^{2}\right)}{Z_{out}+\left(Z_{in}\cdot {V_{out}}^{2}\right)-(2\cdot Z_{in}\cdot V_{out})}$$

(21) $$R_{shunt-out}=\frac{V_{out}}{\frac{1}{Z_{in}}-\frac{V_{out}}{Z_{out}}-\frac{1}{{shunt}_{in}}}$$

(22) $$R_{series}=\frac{\left(1-V_{out}\right)\;\cdot \;\left(Z_{in}\cdot {shunt}_{in}\right)}{{shunt}_{in}-Z_{in}\;}$$

In a real power line, PD sources are generated somewhere in the dielectric between the main conductor and the cable screen or GIS enclosure. In these cases, the pulses first propagate through the healthy part of the dielectric until they reach the active or screen/enclosure of the affected element and then through the transmission line. However, when the PD source is simulated with the ASG, the healthy part of the dielectrics cannot be considered. In this case, the pulses are injected directly between the active and the screen/enclosure and then propagate through the line. This approach to injecting the pulses can be considered suitable for the following reason. In the case of a real insulation defect, at the position where the PD source is located, the capacity of the defective dielectric is much smaller than is the one in the series of the healthy dielectric, so the former prevails, and the latter can be neglected. Consequently, for practical purposes, the method of simulating PD sources with the ASG, where only the capacity of the defective dielectric is considered, is adequate. The previous consideration is the same as that accepted in the calibration process of a PD measurement, when the calibrator is connected to the test object.

The PD time series to be injected with the ASG were previously measured in a controlled way in cable accessories and test cells where real insulation defects were generated. Pulsating noise signals characteristic of electrical grids can also be generated with the ASG to simulate this type of noise condition. These signals are injected in the test platform in the same way as are the PD pulses time series.

### 2.7. Background Noise Signal Simulation

**Figure 13:**
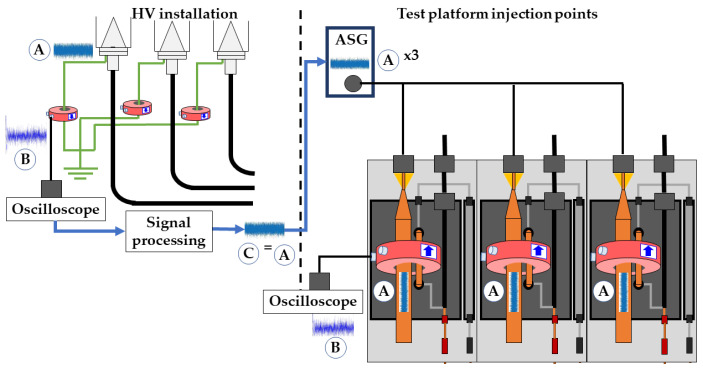
Online noise measurement, signal processing, noise generation, and noise measurement in the test platform.

To simulate the same background noise conditions as those seen in a real installation, the following procedure was established. In the first step, the noise signals to be injected were previously measured online with HFCT sensors in real cable systems. The bandwidth of these sensors was up to 80 MHz. The original and measured noise time series are identified with the letters A and B, respectively, in Figure 13. In the second step, the original signals, identified with letter C, were recovered by signal reconstruction, applying the method explained in [23]. This method was developed experimentally through sensor characterization. After this step, the noise time series were prepared for generation with the ASG.

**Figure 14:**
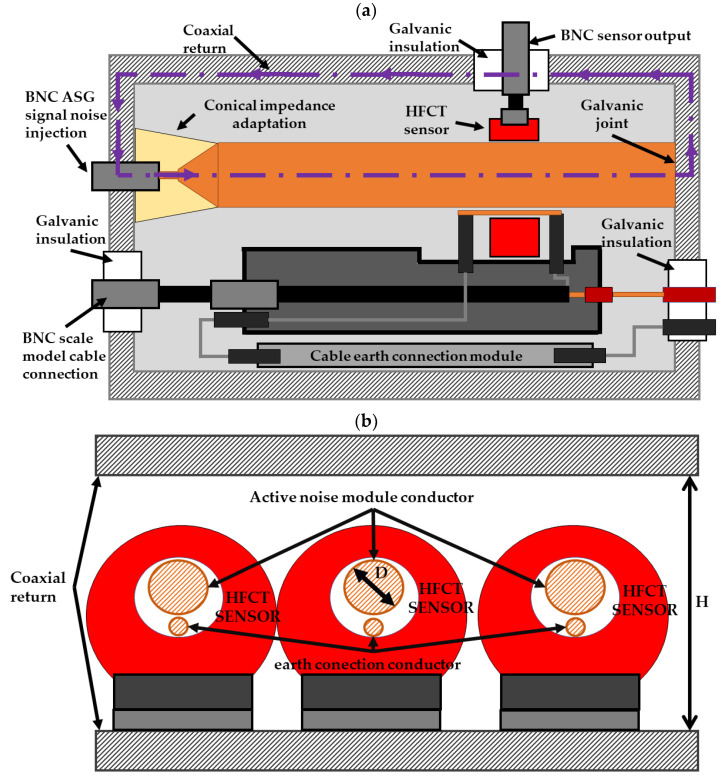
Module designed for the background noise injection. (**a**) Longitudinal view and (**b**) transversal view.

In the third step, the noise signal time series are injected in the test platform to be measured using the HFCT sensors. In this process, to avoid changes in the noise signal waveform at the injection points, the coaxiality in the transmission medium must be maintained. Thus, a galvanically isolated module that wraps the three sensors of each measuring position was designed. The noise signals are conducted through the three sensors individually (by means of three different conductors), offering the possibility of injecting the same or a different noise time series in each phase (see Figure 13 and Figure 14). With this configuration, changes in the signal waveform that might occur if a unique conductor were to be passed through the three sensors are prevented. To avoid reflections, the noise module must have the same characteristic impedance as the ASG (50 Ω). This impedance value is achieved via the appropriate design of the module dimensions. First, the ideal characteristic impedance (*Z_k_*) is calculated using Equation (23) [34] given that the active conductor diameter implemented for the noise injection is zero.

(23) $$Z_{k}=\frac{4\cdot H}{\pi \cdot \;e^{\frac{Z_{0}\cdot \sqrt{\varepsilon_{r}}\;}{60}}}$$

*Z*_0_ is the characteristic impedance (50 Ω), *ε_r_* is the relative permeability of air (1.01), and *H* is the inner module height, set as 120 mm for an HFCT sensor height of up to 120 mm. Then, the diameter of the active conductor *D* can be determined by applying Equation (24).

(24) $$Z_{k}=\frac{2}{1-\frac{D}{H}}\cdot ln\left(\frac{1}{1-\frac{D}{H}}+1\right)-\left(\frac{1}{1-\frac{D}{H}}-1\right)\cdot ln\left(\frac{1}{{\left(1-\frac{D}{H}\right)}^{2}}-1\right)$$

Setting the module height to accommodate sensors with an outer diameter of up to 120 mm results in a conductor with a diameter of 55 mm. With this design, a convergence in the measured signals B (see Figure 13) is obtained in the time and frequency domain.

## 3. Test Platform Validation

To validate the test platform, the similarities between the pulses’ behaviors here and in a real installation of 66 kV were analyzed. The convergence of the pulses was checked in the following parts of both installations:

- In the cable system, where the cable design was validated;
- In the measuring points, where the cable–GIS connection and the earth connection designs were validated.

### 3.1. Cable System Validation

In the design process presented in Section 2.3, the total cable length set in the test platform to simulate a real 66 kV cable system of 1740 m was 300 m.

**Figure 15:**
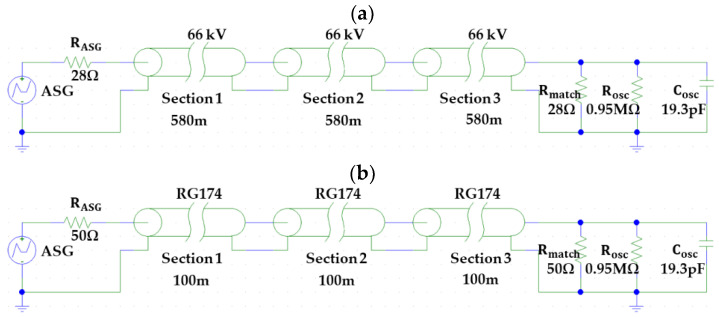
Layout of the modeled cables. (**a**) Cable of 66 kV of a real installation and (**b**) the RG 174 cable of the test platform.

Given the impossibility of performing measurements in a real 66 kV cable section of 1740 m and the lack of availability in the design process of a real RG 174 cable of 300 m, the validation was carried out by modeling both cables with the required lengths using the software PSPICE (version 9.1). Figure 15 shows the layouts of both cables. The values of the distributed cable parameters considered for the models were obtained via the theoretical and practical development presented in Appendix A.

The similarity between the pulse behaviors in both cables was checked by injecting a reference pulse with the ASG at the beginning of the cable and measuring it in the positions shown in Table 3. The measuring point shown in Figure 15 was positioned at the end of the cables. The pulse to be injected was determined by Equations (15) and (16) in Section 2.5.2.

**Figure 16:**
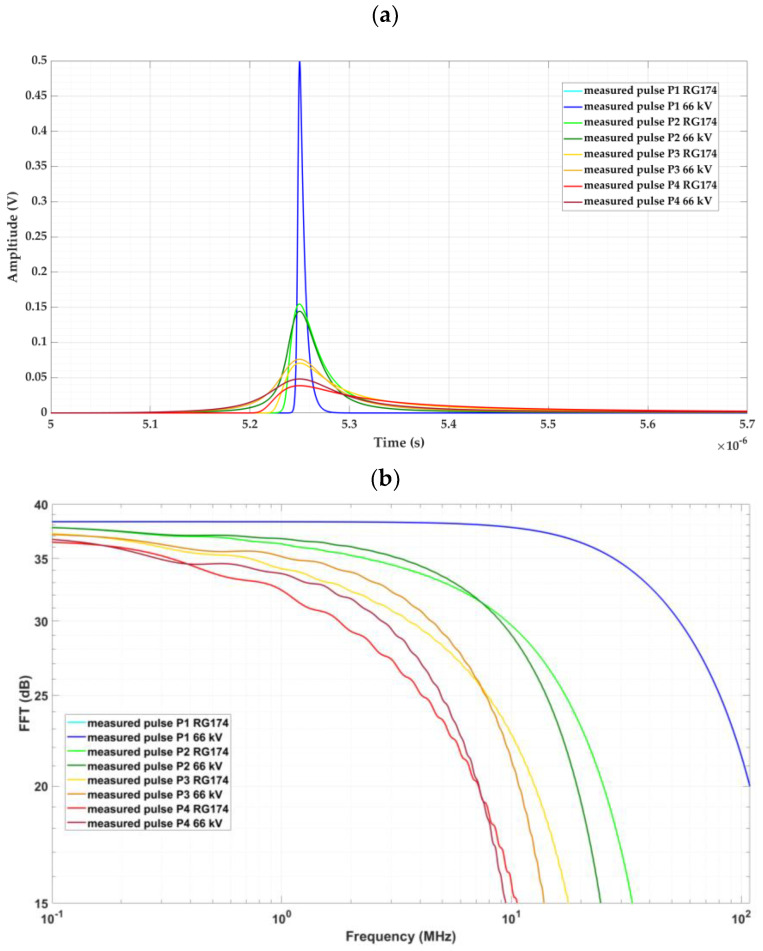
Pulse behavior of pulses measured at different positions. (**a**) Signals in the time domain and (**b**) signals in the frequency domain.

Figure 16 shows the waveform and frequency spectrum of the pulses measured in both installations at the distances indicated in Table 3.

To check the similarity of the pulses measured at the same positions, the following parameters were considered: peak voltage, energy, and frequency limit above which the pulse loses 90% of its energy (see Table 3).

After analyzing the results shown in the third column, as expected, we found that the convergence of the pulses was better when the distance traveled was shorter. In all cases, the convergence was higher than 85%. Therefore, it can be concluded that the cable system designed for the test platform satisfactorily simulates a real cable system of 66 kV.

### 3.2. Cable–GIS Connection and Earth Connection Validation

For the validation of the cable–GIS connection and earth connection, the similarity between the pulse behaviors in the cable–earth connections of the real 66 kV installation and in the test platform was checked. For the pulse comparison, a laboratory setup consisting of two meters of the 66 kV cable connected to a 66 kV GIS section was used. The reference pulse defined by Equations (15) and (16) was injected at the end of the 66 kV cable and at the end of a one-meter-long RG 174 cable connected to the GIS element of the test platform.

The pulses were measured with HFCT sensors located at the cable–earth connections of both setups. The bandwidth of this sensor ranged from 100 kHz to 80 MHz. The cable length of the earth connection in both installations was 10 m, in accordance with the distances considered in the design process presented in Section 2.5.2.

**Figure 17:**
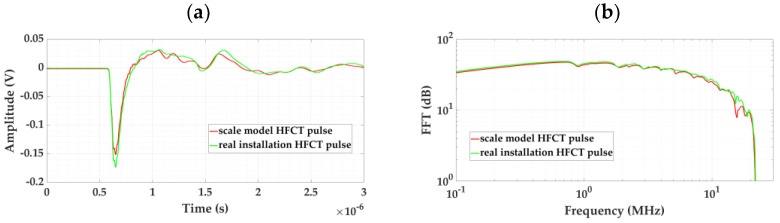
Pulses measured with the HFCT sensors in the 66 kV cable–GIS setup and in the test platform. (**a**) Signals in the time domain and (**b**) signals in the frequency domain.

Figure 17 shows the pulses measured with the HFCT sensors in the two real installations, together with their frequency spectra. 

To determine the similarity of the pulses, again, the following parameters were considered: peak voltage, energy, and frequency limit above which the pulse loses 90% of its energy (see Table 4).

When analyzing the results shown in the third column, we found that, in all cases, the convergence was higher than 88%. Therefore, it can be concluded that the cable–GIS connection and earth connection in the test platform satisfactorily simulate the cable–GIS connection and earth connection of a real 66 kV installation.

## 4. Conclusions

An affordable scale modular test platform that simulates HV installations for the adequate and repetitive or standardized characterization of PD-measuring systems was developed in this study. The availability of this reference test platform provides a solution to the difficulties encountered by technology developers and electrical companies when they characterize PD-measuring systems using complex laboratory setups or real on-site installations. The use of laboratory setups is costly, and real installations are generally not available; furthermore, in both cases, the influences of the noise conditions on the measurements are not controlled. The performance of characterization tests with this test platform in shielded chambers enables the control of noise conditions.

In the design process, the following technical aspects were considered:

- The signals’ transmission, attenuation, distortion, reflection, and propagation speed;
- The signals’ behavior in the measuring points;
- The best way to simulate the insulation defects and the electrical noise conditions;
- The sensor coupling to reproduce real on-site measuring conditions.

The test platform’s functionality was validated by checking the convergence of the measured signals within it with those of a real HV installation.

With the designed platform, it is possible to perform all kinds of tests for the characterization of the PD-measuring and -monitoring system’s functionalities, such as those related to their capacity to reject noise signals, detect PD with the required sensitivity, detect the phase(s) where an insulation defect is present, discriminate the presence of more than one defect, locate them, identify them, identify the affected element in the installation, and generate an alarm when potentially hazardous defects are identified.

It is important to indicate that due to its modular design, the developed platform can be used for the characterization of PD-measuring systems operating off-line. The modular design also enables the extension of the platform to simulate more complex installations for the characterization of measuring systems operating in other environments and with other sensors in addition to the HFCTs. However, to perform characterizations in these complementary test platforms, further studies must be performed to simulate the same measuring conditions as found in on-site installations. The authors are currently working on new complementary platforms.

The scale modular design ensures the permanent availability and easy portability of this reference test platform such that the characterization of measuring systems can be carried out at anytime and anywhere in the world. This enables the possibility of performing intercomparisons among different technologies.

Furthermore, apart from its use in the research and industrial fields, it can be used in the training of specialist technicians in PD measurements and in training courses for electrical engineers.

An example of the use of this test platform for the characterization of PD-measuring systems will be presented by the authors in a forthcoming publication.

## 5. Patents

The developments associated with the test platform presented in this research article are protected by patent application no. 202331099 and reference no. P-102092, filed with the Spanish Patent and Trademark Office.

## Figures and Tables

**Table 1:** Assessment of the MIL-C-17 coaxial cables adequate for the scale model.

Technical Characteristic		Cable Type			
		LLF 240	RG 58	RG 59	RG 174
Cost	Value (p.u.)	1	0.33	0.28	0.31
	Rating	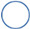	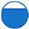	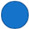	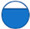
Weight	Value (p.u.)	0.99	0.72	1	0.21
	Rating	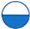	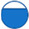	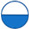	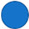
Volume	Value (p.u.)	0.99	0.81	1	0.44
	Rating	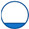	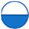	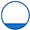	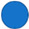
Characteristic impedance	Value (Ω)	50	50	75	50
	Rating	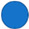	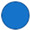	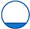	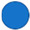
Attenuation	Value (p.u.)	0.33	0.43	0.29	1
	Rating	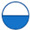	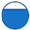	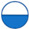	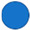
Propagation speed	Value	252	198	198	198
	Rating	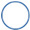	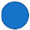	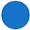	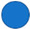
Total score (points)		9	19	14	23

**Table 2:** Additional distances traveled by the reflected pulses with insulation defects simulated at points 1, 2, 3, and 4 and measured at position 1.

	Defect Location			
	Point 1 (Line Start)	Point 2 (Joint 1)	Point 3 (Joint 2)	Point 4 (Line End)
Additional distance traveled by the reflected pulses	6A	4A	2A	6A

**Table 3:** Results obtained for the pulses measured in both cable systems.

Measuring Point	Cable Model								% Convergence		
	66 kV				RG 174						
	Traveled Distance (m)	Vpeak (mV)	Fmax (MHz)	Energy (v^2^·Hz)	Traveled Distance (m)	Vpeak (mV)	Fmax (MHz)	Energy (v^2^·Hz)	Vpeak	Fmax	Energy
1	0	500	70.2	4.97	0	500	70.2	4.97	100%	100%	100%
2	588	145.9	23.1	1.17	100	154.8	26.8	1.19	94%	86%	98%
3	1.176	76.2	13.1	0.53	200	70.7	15.2	0.48	93%	86%	91%
4	1.764	47.1	8.1	0.32	300	39.8	9.5	0.27	85%	85%	85%

**Table 4:** Results obtained for the measured pulses in the 66 kV laboratory setup and in the test platform.

66 kV Cable–GIS Laboratory Setup			Test Platform			% Convergence		
Vpeak (mV)	Fmax (MHz)	Energy (v^2^·Hz)	Vpeak (mV)	Fmax (MHz)	Energy (v^2^·Hz)	Vpeak	Fmax	Energy
173.1	8.97	22.1 × 10^−3^	152.8	8.57	21.2 × 10^−3^	88%	96%	96%

**Figure A1:**
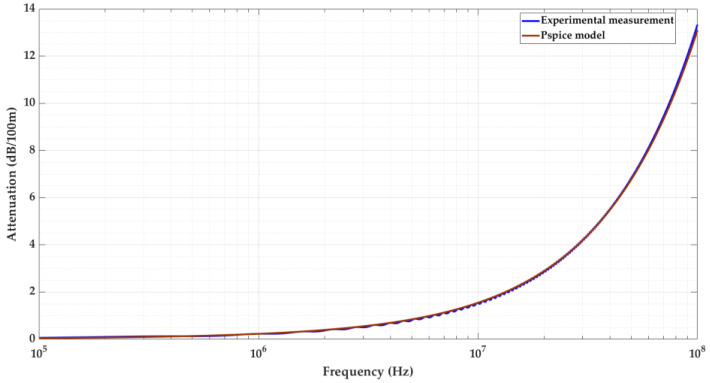
Frequency response of the modeled and real 66 kV cable sections.

**Figure A2:**
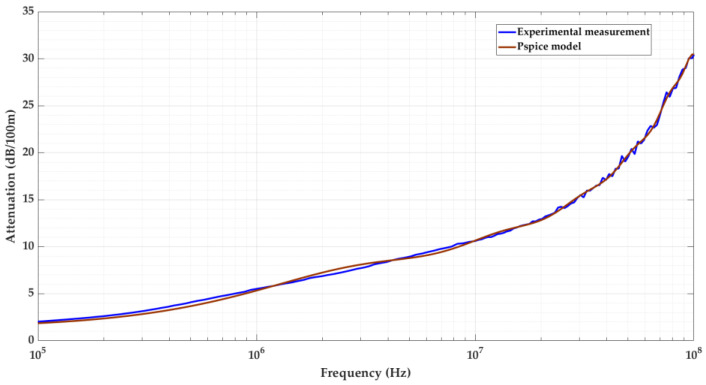
Frequency response of the modeled and real RG 174 cable section.

## Data Availability

Data are contained within the article.

## Appendix A. Determination of the Distributed Parameters of the 66 kV Cable, RG 174 Cable, 66 kV GIS, and Test Platform GIS for Modeling

In the study of transmission lines [35,36], there are different ways to model cables and GIS; for example, by applying the finite difference time domain theory [37] or the distributed parameters theory [38,39]. In this study, to model a cable of 66 kV, the RG 174 cable, a 66 kV GIS, and a test platform GIS, the latter theory was adopted. Following this method, these elements were studied by considering infinitesimal segments (see Figure 8 and Figure 10).

The distributed parameters of these elements can be determined from their physical structure [26,38,40]. Thus, the distributed resistance (*R_d_*), inductance (*L_d_*) and conductance (*G_d_*) can be calculated using Equations (A1) and (A2) [26] and Equation (A3) [38,40], respectively. The distributed capacitance of the 66 kV cable (*C_d_*) is calculated using Equation (A4), with the following capacitances of the cable layers being added from the active to the screen: internal semiconductor layer (A5), insulation (A6) and external semiconductor layer (A7) [26,38,40]. For the RG 174 cable and both GISs, only the insulation capacitance (A6) is considered since in these elements, there are no semiconductor layers. In these equations, the following parameters are taken into account: the vacuum permeability (*µ*_0_), the active conductor permeability (*µ_act_*), its conductivity (*σ_act_*), its radius (*r_1_*), the shield radius (*r*_2_), the insulation conductivity (*σ_ins_*), the inner semiconductor layer’s diameter (*d*_sc1_), the outer semiconductor layer’s diameter (*d_sc_*_2_), the vacuum dielectric constant ($\varepsilon_{0}$) and the dielectric constants of the main insulation ($\varepsilon_{ins}(f)$), the inner semiconductor layer ($\varepsilon_{sc1}(f))$, and the outer semiconductor layer ($\varepsilon_{sc2}(f)$, which are frequency-dependent.

(A1) $$R_{d}=\sqrt{\frac{2\cdot \;\pi \cdot f\cdot \mu_{0}\cdot \mu_{act}}{2\cdot \sigma_{act}}\;}\cdot \left(\frac{1}{r_{1}}+\frac{1}{r_{2}}\right)$$

(A2) $$L_{d}=\frac{\mu_{0}\cdot \mu_{act}}{2\cdot \;\pi \;}\cdot ln\left(\frac{r_{2}}{r_{1}}\right)$$

(A3) $$G_{d}=\frac{2\cdot \pi \cdot \sigma_{ins}}{ln\left(\frac{r_{1}+d_{sc1}+{d_{ins}+d}_{sc2}}{r_{1}}\right)}$$

(A4) $$C_{d}=\frac{1}{\frac{1}{{C_{d}}_{sc1}}+\frac{1}{{C_{d}}_{ins}}+\frac{1}{{C_{d}}_{sc2}}}$$

(A5) $${C_{d}}_{sc1}=\frac{2\cdot \pi \cdot \varepsilon_{0}\cdot \varepsilon_{sc1}(f)}{ln\left(\frac{r_{1}+d_{sc1}}{r_{1}}\right)}$$

(A6) $${C_{d}}_{ins}=\frac{2\cdot \pi \cdot \varepsilon_{0}\cdot \varepsilon_{ins}(f)}{ln\left(\frac{r_{1}+d_{sc1}+d_{ins}}{r_{1}+d_{sc1}}\right)}$$

(A7) $${C_{d}}_{sc2}=\frac{2\cdot \pi \cdot \varepsilon_{0}\cdot \varepsilon_{sc2}(f)}{ln\left(\frac{r_{1}+d_{sc1}+d_{ins}+d_{sc2}}{r_{1}+d_{sc1}+d_{ins}}\right)}$$

Table A1 shows the values calculated for the distributed parameters of the four elements.

**Table A1:** Parameters and values calculated with the estimation of the distributed parameters.

	*R_d_* (µΩ/m)	*L_d_* (µH/m)	*G_d_* (pS/m)	*C_d_* (pF/m)
Test platform (Cable RG 174)	127.82	0.25	0.1	101
Real installation (Cable 66 kV)	1.133	0.16	2.77	211
Tet platform GIS	-	0.39	-	45
66 kV GIS	-	0.20	-	67

Using the values of the distributed parameters calculated for a cable of 66 kV, a section of 10 m was modeled with PSPICE software (version 9.1). To check that the values used in the model were correct, a frequency sweep was performed in the modeled cable and in a real 66 kV cable section of the same length. The results are shown in Figure A1. The convergence obtained indicates that the values calculated for the model are correct.

Likewise, using the values of the distributed parameters calculated for the RG 174 cable, a cable section of 100 m was modeled with the same software. To verify that the values used in the model were correct, a frequency sweep was performed on the simulated cable and on a real RG 174 cable of the same length. The results are shown in Figure A2. The convergence obtained indicates that the values calculated for the model are correct.

In the cases of both GISs, for the simulation carried out in Section 2.5.2, only their characteristic impedances were of interest. These impedances were calculated using Equation (12) for the distributed parameters *L_d_* and *C_d_* shown in Table A1. The resulting characteristic impedance for the 66 kV GIS matched with the value estimated by the GIS manufacturers.

## References

[1] Haddad A., Warne D.F. (2007). Partial Discharges and Their Measurement. Advances in High Voltage Engineering.

[2] Sahoo R., Karmakar S. (2023). Investigation of electrical tree growth characteristics and partial discharge pattern analysis using deep neural network. Electr. Power Syst. Res..

[3] Wester F. (2004). Condition Assessment of Power Cables Using PD Diagnosis at Damped AC Voltages. Ph.D. Thesis.

[4] Álvarez F., Garnacho F., Ortego J., Sánchez-Urán M.A. (2015). Application of HFCT and UHF sensors in on-line partial discharge measurements for insulation diagnosis of high voltage equipment. Sensors.

[5] Tian Y., Lewin P., Davies A. (2002). Comparison of On-line Partial Discharge Detection Methods for HV Cable Joints. IEEE Trans. Dielectr. Electr. Insul..

[6] Álvarez F., Ortego J., Garnacho F., Sanchez-Urán M.A. Advanced techniques for on-line PD measurements in high voltage systems. Proceedings of the 2014 IEEE International Conference on High Voltage Engineering and Application (ICHVE).

[7] Steennis F., Wagenaars P., van der Wielen P., Wouters P., Li Y., Broersma T., Harmsen D., Bleeker P. (2016). Guarding MV cables on-line: With travelling wave based temperature monitoring, fault location, PD location and PD related remaining life aspects. IEEE Trans. Dielectr. Electr. Insul..

[8] Montanari G.C., Cavallini A., Pasini G. (2009). A Method for Detecting, Identifying and Locating Partial Discharges Occurring in a Discharge Site along an Electric Apparatus. Patent.

[9] Cavallini A., Montanari G.C., Puletti F., Contin A. (2005). A new methodology for the identification of PD in electrical apparatus: Properties and applications. IEEE Trans. Dielectr. Electr. Insul..

[10] Stone G. (1991). Partial discharge-part VII. Practical techniques for measuring PD in operating equipment. IEEE Electr. Insul. Mag..

[11] Koltunowicz W., Plath R. (2008). Synchronous multi-channel PD measurements. IEEE Trans. Dielectr. Electr. Insul..

[12] Ardila-Rey J.A., Rojas-Moreno M.V., Martínez-Tarifa J.M., Robles G. (2014). Inductive sensor performance in partial discharges and noise separation by means of spectral power ratios. Sensors.

[13] Wang Y., Chen P., Zhao Y., Sun Y. (2022). A Denoising Method for Mining Cable PD Signal Based on Genetic Algorithm Optimization of VMD and Wavelet Threshold. Sensors.

[14] Álvarez F., Arcones E., Garnacho F., Ramírez A., Ortego J. Efficient PD Monitoring of HV Electrical Systems Using HFCT Sensors. Proceedings of the 2018 IEEE International Conference on High Voltage Engineering and Application (ICHVE).

[15] Renforth L.A., Giussani R., Mendiola M.T., Dodd L. (2019). Online Partial Discharge Insulation Condition Monitoring of Complete High-Voltage Networks. IEEE Trans. Indust. Applic..

[16] Rodrigo A., Castro L.C., Harmsen D.A., Muñoz F.A. (2018). A new design of a test platform for testing multiple partial discharge sources. Int. J. Electr. Power Energy Syst..

[17] Garnacho F., Ramírez Á., Álvarez F., Arcones E., Vera C.A. Characterization of partial discharge measuring instruments by the generation of reference insulation defects in an experimental setup. Proceedings of the 2018 IEEE 2nd International Conference on Dielectrics (ICD).

[18] Hu Y., Chiampi M., Crotti G. (2015). Characterisation system for the evaluation of digital partial discharge measuring instruments. IET Sci. Meas. Technol..

[19] Hu Y., Chiampi M., Crotti G., Sardi A. Setting-up of a characterization system for digital PD measuring instruments. Proceedings of the 2010 Conference on Precision Electromagnetic Measurements (CPEM).

[20] Khamlichi A., Garnacho F., Simón P. (2023). New Synthetic Partial Discharge Calibrator for Qualification of Partial Discharge Analyzers for Insulation Diagnosis of HVDC and HVAC Grids. Sensors.

[21] Vera C., Garnacho F., Klüss J., Mier C., Álvarez F., Lahti K., Khamlichi A., Elg A.-P., Rodrigo Mor A., Arcones E. (2023). Validation of a Qualification Procedure Applied to the Verification of Partial Discharge Analysers Used for HVDC or HVAC Networks. Appl. Sci..

[22] (1990). USA Government, Military Specification: Cables, Radio Frequency, Flexible and Semirigid, Generation Specification for, MIL-C-17G (AMENDMENT 3). https://nepp.nasa.gov/docuploads/96D38FB4-6F63-45A5-8CB5ABCA633430EB/MIL-C-17.pdf.

[23] Paschotta R. Article on “Propagation Constant” in the RP Photonics Encyclopedia. https://www.rp-photonics.com/propagation_constant.html.

[24] Fiore J.M. (2023). AC Electrical Circuit Analysis: A Practical Approach.

[25] Martinez Velasco J.A. (2008). Coordinación de Aislamiento en Redes Elétricas de Alta Tensión.

[26] Fritsch M., Wolter M. (2022). Transmission Model of Partial Discharges on Medium Voltage Cables. IEEE Trans. Power Deliv..

[27] Shi Q., Tröltzsch U., Kanoun O. Analysis of the parameters of a lossy coaxial cable for cable fault location. Proceedings of the Eighth International Multi-Conference on Systems, Signals & Devices.

[28] Paul C.R. (2012). Transmission Lines: Physical Dimensions vs. Electric Dimensions. Transmission Lines in Digital Systems for EMC Practitioners.

[29] Darwish A., Refaat S.S., Toliyat H.A., Abu-Rub H. (2019). On the Electromagnetic Wave Behavior Due to Partial Discharge in Gas Insulated Switchgears: State-of-Art Review. IEEE Access.

[30] Tanaka Y., Baba Y. (2023). Development of a Method for Calculating the Capacitance of Cables Installed in a Rectangular Tunnel Taking Account of the Permittivity of the Outer Sheath. IEEE Trans. Electromagn. Compat..

[31] Clements C., Paul C.R., Adams A.T. (1975). Computation of the Capacitance Matrix for Systems of Dielectric-Coated Cylindrical Conductors. IEEE Trans. Electromagn. Compat..

[32] Giri D.V., Prather W.D. (2013). High-Altitude Electromagnetic Pulse (HEMP) Risetime Evolution of Technology and Standards Exclusively for E1 Environment. IEEE Trans. Electromagn. Compat..

[33] Vizmuller P. (1995). RF Design Guide Systems, Circuits and Equations.

[34] Hammood M.K. (2012). Impedance of Stripline. Tikrit J. Pure Sci..

[35] Pozar D.M. (2005). Microwave Engineering.

[36] Hayt W.H., Buck J.A. (2006). Engineering Electromagnetics.

[37] Hu X., Siew W.H., Judd M.D., Reid A.J., Sheng B. (2019). Modeling of High-Frequency Current Transformer Based Partial Discharge Detection in High-Voltage Cables. IEEE Trans. Power Deliv..

[38] Khamlichi A., Garnacho F., Álvarez F. Cable model for partial discharge measurements. Proceedings of the 2016 IEEE International Conference on Dielectrics (ICD).

[39] Tozzi M., Cavallini A., Montanari G.C., Burbui G.L.G. (2008). PD detection in extruded power cables: An approximate propagation model. IEEE Trans. Dielectr. Electr. Insul..

[40] Thayoob Y.H.M., Ariffin A.M., Sulaiman S. Analysis of high frequency wave propagation characteristics in medium voltage XLPE cable model. Proceedings of the 2010 International Conference on Computer Applications and Industrial Electronics.

